# Comparison of image quality between simultaneous multi-slice single-shot EPI and readout-segmented EPI in diffusion-weighted imaging of prostate cancer: a retrospective study

**DOI:** 10.3389/fonc.2026.1727062

**Published:** 2026-02-11

**Authors:** Tianqi Zhang, Jie Chen, Meng Li, Aibin Shen

**Affiliations:** 1 Department of Computed Tomograpgy/Magnetic Resonance (CT/MR), Xingtai People’s Hospital, Xingtai, Hebei, China; 2 Department of Oncology II, Xingtai People’s Hospital, Xingtai, Hebei, China; 3 Department of Imaging, Cangzhou People’s Hospital, Cangzhou, China; 4 Department of Ultrasonography, Tangshan City Fengnan District Hospital, Tangshan, Hebei, China

**Keywords:** diagnostic efficiency, diffusion weighted imaging, image quality, prostate cancer, readout-segmented EPI, simultaneous multi-slice

## Abstract

**Background:**

Simultaneous multi-slice (SMS) has received a lot of attention, but there is a lack of comparative studies on the image quality and diagnostic efficacy of simultaneous multi-slice single-shot echo planar imaging (SMS+SS-EPI) versus readout-segmented echo planar imaging (RS-EPI) in diffusion-weighted imaging (DWI) of prostate cancer.

**Objectives:**

Comparison of image quality and diagnostic efficacy of SMS+SS-EPI and RS-EPI in DWI of prostate cancer.

**Methods:**

A retrospective study included 100 patients who underwent magnetic resonance imaging (MRI) between January and August 2025 (48 cases with SMS+SS-EPI and 52 cases with RS-EPI). Two radiologists performed blinded 5-point subjective scoring and measured lesion signal-to-noise ratio (SNR), contrast-to-noise ratio (CNR), contrast (C), and apparent diffusion coefficient (ADC) values. Using transperineal 12+X needle biopsy pathology as the gold standard, receiver operating characteristic (ROC) curves were plotted and ADC diagnostic performance was compared.

**Results:**

The RS-EPI group demonstrated superior performance in clarity (4.37 ± 0.69 vs. 3.98 ± 0.79), anatomical distortion (4.06 ± 0.80 vs. 3.69 ± 0.78), sharpness (4.04 ± 0.82 vs. 3.58 ± 0.74), detail display (4.21 ± 0.72 vs. 3.75 ± 0.70), and overall quality (4.33 ± 0.68 vs. 3.88 ± 0.70) were better than those of the SMS+SS-EPI group (P<0.001). The SNR (57.65 ± 7.84 vs. 50.45 ± 6.56, P < 0.001) and CNR (4.58 ± 0.75 vs. 4.16 ± 0.73, P = 0.005) were significantly higher than those in the RS-EPI group, while C (6.43 ± 1.06 vs. 6.32 ± 1.02, P = 0.578) and ADC (0.90 ± 0.23 vs. 0.87 ± 0.21, P = 0.448) values showed no statistically significant differences. Additionally, the area under curve (AUC) values for diagnosing prostate cancer based on ADC in the two groups were 0.925 and 0.933, respectively, indicating statistical equivalence in diagnostic performance (z=0.462, P = 0.644). However, the SMS+SS-EPI group demonstrated a significantly shorter acquisition time (1 min 50 s vs. 3 min 43 s).

**Conclusion:**

RS-EPI delivers superior subjective image quality, facilitating detailed anatomical assessment. SMS+SS-EPI provides higher SNR and CNR, significantly reducing scan time while maintaining comparable diagnostic performance based on ADC values. Sequence selection should be guided by clinical requirements.

## 1 Introduction

Prostate cancer is the second most common malignant tumor among men worldwide and poses a major threat to men’s health (1–3). In recent years, the incidence and mortality rates of prostate cancer have been on the rise in China, and early detection and accurate diagnosis are crucial for improving patient prognosis (4–6). Magnetic resonance imaging (MRI) plays a key role in the diagnosis and management of prostate cancer due to its excellent soft tissue contrast and functional imaging capabilities (7–9). Among various MRI sequences, diffusion-weighted imaging (DWI) has gained wide clinical acceptance for its ability to non-invasively reflect tissue microstructure (10, 11). DWI has become an essential component of the prostate imaging reporting and data system (PI-RADS) for lesion detection and characterization (12).

The traditional single-shot echo-planar imaging (SS-EPI) sequence is the most commonly used DWI sequence in clinical practice. Due to its convoluted k-space filling method, long echo chain, and correspondingly low bandwidth in the phase encoding direction, it is prone to producing significant susceptibility artifacts, geometric distortions, and image blurring, which compromise the accuracy of parameter measurements (13–15). Readout-segmented echo planar imaging (RS-EPI) can reduce echo spacing and improve DWI image quality. However, RS-EPI requires segmented readout in the readout direction, followed by integration into a complete k-space, resulting in longer imaging times (16, 17). With technological advancements, simultaneous multi-slice (SMS) technology has garnered significant attention. By combining multiple radiofrequency pulses into a composite pulse, SMS enables simultaneous excitation and acquisition of multiple slices, significantly reducing repetition time (TR) and scan time (ST) (18, 19). Unlike parallel imaging techniques, SMS avoids signal-to-noise ratio (SNR) loss due to data under sampling (20–22). Although SMS-enhanced DWI has shown promising results in neuroimaging, breast, and rectal applications (23, 24). However, there is currently no systematic evidence specifically comparing SMS+SS-EPI with RS-EPI for prostate diffusion-weighted imaging (DWI). In particular, there is a lack of comprehensive evaluations linking quantitative image quality metrics such as SNR and CNR with performance parameters like ADC values and diagnostic efficacy. This retrospective study aims to provide the first direct comparison between the SMS+SS-EPI and RS-EPI sequences in diffusion-weighted imaging (DWI) for prostate cancer. Not only to evaluate subjective and objective image quality, but more importantly, to compare the diagnostic performance of the SMS+SS-EPI sequence versus the RS-EPI sequence through ADC-based ROC analysis. It is hoped that the findings will provide valuable clinical evidence for the widespread application of SMS technology in prostate cancer imaging, thereby promoting early diagnosis and more effective treatment strategies.

## 2 Materials and methods

### 2.1 Patients

**Figure 1:**
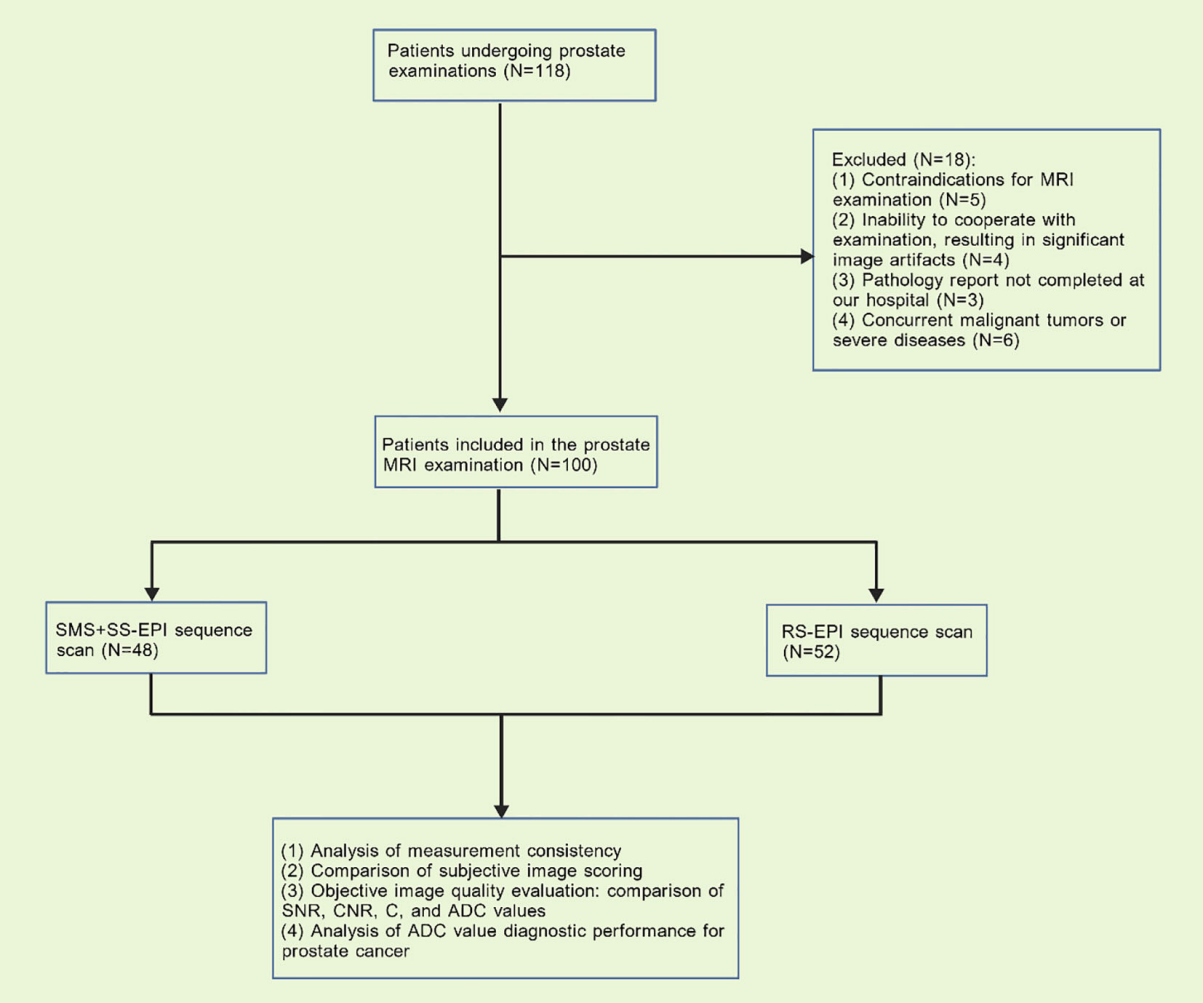
Research flowchart.

The sample size was calculated *a priori* using G Power 3.1 software for an independent samples t-test, based on an anticipated large effect size (d=0.8) for the primary comparisons of image quality parameters (subjective scores and key objective metrics) between the two sequences, with α=0.05 and power (1-β) of 0.90. This yielded a minimum total sample size of 68. Data were retrospectively collected from 118 patients who underwent prostate examinations at our hospital between January 2025 and August 2025. Eighteen patients were excluded due to non-compliance with inclusion criteria. Ultimately, 100 patients who underwent prostate MRI were included in the analysis. Among these, 48 patients were scanned using the SMS+SS-EPI sequence, and 52 patients were scanned using the RS-EPI sequence (**Figure 1**). This study was approved by the Ethics Committee of Xingtai People’s Hospital (Approval No. 2025【037】).

### 2.2 Inclusion and exclusion criteria

Inclusion Criteria: (1) No history of non-surgical treatments such as hormone therapy or radiotherapy prior to prostate MRI examination; (2) No prior prostate biopsy; (3) Complete clinical documentation; (4) Voluntary signing of informed consent. Exclusion Criteria: (1) Presence of MRI examination contraindications; (2) Inability to cooperate with the examination resulting in significant image artifacts; (3) Pathology report not completed at our institution; (4) Concurrent other malignancies or severe diseases.

### 2.3 MRI examination method

All examinations were performed using either a Siemens Vida 3.0T MRI. Prior to scanning, patients were instructed to empty their bowels as much as possible and moderately fill their bladders. Scans were acquired in the supine position using a pelvic phased array coil. All patients underwent axial, coronal, and sagittal fast spin-echo T2-weighted imaging (T2WI) sequences, axial fat-suppressed T2WI, and fast spin-echo T1-weighted imaging (T1WI) sequences. RS-EPI sequence parameters: repetition time (TR) 4700ms, echo time (TE) 62/99ms, echo gap 0.30ms, field of view (FOV) 206×206, matrix 128×128, slice thickness 3mm, non-gap scanning, segmented readout 7, b-values 50, 1000/mm². SMS+SS-EPI sequence parameters: TR 4900ms, TE 78ms, echo gap 0.94ms, FOV 220×176, matrix 90×90, slice thickness 3mm, non-gap scanning, b-values 50, 1000/mm², SMS acceleration factor 2. It is noteworthy that the RS-EPI parameters are set to minimize distortion and maximize spatial detail, while the SMS+SS-EPI parameters are optimized to utilize synchronous multi-slice acceleration to shorten scan time. Although this results in differences in FOV and matrix, this comparison aims to evaluate the performance of these sequences in actual clinical applications.

### 2.4 Prostate biopsy and histopathological diagnosis

Prostate biopsy is performed by a senior urologist using a transperineal systematic saturation biopsy combined with MRI-guided targeted biopsy of suspicious lesions. The systematic saturation biopsy employs the internationally recognized 12-site approach, while the targeted biopsy of lesions involves approximately 1–3 additional needles. Specimens are immediately sent to the pathology department for paraffin embedding and staining. A senior pathologist experienced in prostate pathology reviews the slides to determine whether the findings represent benign changes or malignant tumors.

### 2.5 Image evaluation and analysis

Subjective evaluation: Two radiologists with 3 and 5 years of abdominal MRI diagnostic experience, respectively, performed double-blinded, independent readings of the images in both groups twice. A 5-point Likert scale (25, 26), was used for subjective independent scoring of anatomical distortion, clarity, image detail display, and sharpness: 1 point: Severe artifacts and distortion, extremely unclear images, undiagnosable; 2 points: Noticeable artifacts, blurred contours, significant distortion, and poor image clarity; 3 points: Acceptable artifacts, well-defined contours, distinguishable between pathological and normal tissues, and identifiable lesions; 4 points: Mild artifacts, clear contours with slight edge distortion, and good lesion visualization; 5 points: No significant distortion, no artifacts, very clear contours, and excellent lesion display. A lower score indicates poorer image quality.

Objective evaluation: Two radiologists performed analysis on a Siemens dedicated imaging workstation. Regions of interest (ROIs) were manually placed on the largest lesion plane within both DWI and ADC images. On the lesion side, ROIs avoided areas of necrosis or hemorrhage, with ROI size adjusted according to lesion dimensions. To ensure consistent ROI size and positioning between SMS+SS-EPI and RS-EPI sequence DWI images, post-processing software was used to copy and paste ROIs between the two sequences. Evaluation metrics included SNR, CNR, C, and ADC values. Final analysis utilized the average of measurements from both radiologists.

The formulas for SNR, CNR, and C are as follows: SNR = SIROI/SDnoise; CNR = (SIROI - SImuscle)/SDnoise; C = SIROI/SImuscle. Here, SIROI denotes the signal intensity of the lesion region in the DWI image at b = 1000 s/mm², SImuscle refers to the signal intensity of the obturator internus muscle, and SDnoise represents the standard deviation of the background noise signal intensity.

### 2.6 Observation indicators

(1) Analysis of general clinical characteristics. (2) Analysis of inter-observer agreement between two physicians. (3) Comparison of subjective image scoring between RS-EPI and SMS+SS-EPI sequences. (4) Objective evaluation of image quality between RS-EPI and SMS+SS-EPI sequences: Comparison of SNR, CNR, C, and ADC values. (5) Analysis of diagnostic efficacy for prostate cancer using ADC values from RS-EPI and SMS+SS-EPI sequences.

### 2.7 Statistical analysis

Statistical analysis and graphing were performed using SPSS 26.0 and GraphPad Prism 8.0 software. Inter-rater agreement for subjective scoring by two physicians was assessed using Kappa analysis, while inter-observer agreement for measurements was evaluated using intraclass correlation coefficients (ICC). Rank-sum tests were applied to compare subjective scores between RS-EPI and SMS+SS-EPI images. Objective image quality metrics—SNR, CNR, C, and ADC data—were compared using t-tests. Using pathological findings as the gold standard, ROC curves were plotted to evaluate the diagnostic efficacy of lesion ADC values for prostate cancer. The area under the curve (AUC), sensitivity, and specificity were calculated, and Z-tests were performed to compare diagnostic performance between different protocols. P < 0.05 was considered statistically significant.

## 3 Results

### 3.1 Clinical dates

A total of 100 patients undergoing routine prostate MRI examinations were included in the analysis. The SMS+SS-EPI group comprised 48 patients with a mean age of 68.15 ± 6.63 years, while the RS-EPI group included 52 patients with a mean age of 69.04 ± 6.49 years. No statistically significant differences were observed between the two groups in age (P = 0.498), body mass index (P = 0.683), prostate-specific antigen (P = 0.548), prostate volume (P = 0.574), or the number of patients with a biopsy Gleason score ≥ 7 (P = 0.921) (**Table 1**).

**Table 1:** Comparison of the general data of the two groups of patients (mean ± SD or n [%]).

Variable	SMS+SS-EPI (n = 48)	RS-EPI (n = 52)	P value
Age (years)	68.15 ± 6.63	69.04 ± 6.49	0.498
BMI (kg/m²)	24.51 ± 2.14	24.68 ± 2.06	0.683
PSA (ng/mL)	10.06 ± 3.15	9.69 ± 2.95	0.548
Prostate volume (mL)	42.82 ± 10.85	43.97 ± 9.48	0.574
Gleason score ≥ 7	30 (62.50)	32 (61.54)	0.921

BMI, body-mass index; PSA, prostate-specific antigen.

### 3.2 Subjective scoring

The two physicians demonstrated high agreement in their subjective ratings of image quality for SMS+SS EPI and RS-EPI sequence imaging, with Kappa values >0.80 and P<0.001 (**Table 2**). Both SMS+SS EPI and RS-EPI sequences achieved subjective image quality scores ≥3. However, the RS-EPI group demonstrated superior definition (4.37 ± 0.69 vs. 3.98 ± 0.79, P < 0.001), anatomical aberration (4.06 ± 0.80 vs. 3.69 ± 0.78, P < 0.001), image sharpness (4.04 ± 0.82 vs. 3.58 ± 0.74, P < 0.001), show details (4.21 ± 0.72 vs. 3.75 ± 0.70, P < 0.001), and overall image quality (4.33 ± 0.68 vs. 3.88 ± 0.70, P < 0.001) compared to the SMS+SS-EPI group, with statistically significant differences (**Table 3**).

**Table 2:** Consistency analysis of subjective scores for overall image quality.

Sequence	SMS+SS-EPI (n = 48)	RS-EPI (n = 52)
Kappa value	0.858	0.818
Standard error	0.067	0.075
95% CI	0.727 ~ 0.990	0.672 ~ 0.964
*Z* value	7.480	7.013
P value	<0.001	<0.001

**Table 3:** Comparison of subjective scoring of images from two different imaging methods (mean ± SD).

Sequence	SMS+SS-EPI (n = 48)	RS-EPI (n = 52)	P value
Definition	3.98 ± 0.79	4.37 ± 0.69	<0.001
Anatomical aberration	3.69 ± 0.78	4.06 ± 0.80	<0.001
Image sharpness	3.58 ± 0.74	4.04 ± 0.82	<0.001
Show details	3.75 ± 0.70	4.21 ± 0.72	<0.001
Overall image quality	3.88 ± 0.70	4.33 ± 0.68	<0.001

### 3.3 Objective evaluation

The agreement between the SNR (ICC: 0.825, 0.806), CNR (ICC: 0.810, 0.773), C (ICC: 0.800, 0.845), and ADC (ICC: 0.875, 0.858) values measured by the 2 physicians for the SMS+SSEPI and RS- EPI serial imaging was high (**Table 4**). The SNR (57.65 ± 7.84 vs. 50.45 ± 6.56, P < 0.001) and CNR (4.58 ± 0.75 vs. 4.16 ± 0.73, P = 0.005) values of the lesions on the DWI images in the SMS+ SSEPI group were significantly higher than those in the RS- EPI group, while the differences in C (6.43 ± 1.06 vs. 6.32 ± 1.02, P = 0.578) and ADC (0.90 ± 0.23 vs. 0.87 ± 0.21, P = 0.448) values were not statistically significant (**Table 5**). Additionally, the acquisition time for the SMS+SS EPI sequence is 1 minute 50 seconds, while the acquisition time for the RS-EPI sequence is 3 minutes 43 seconds.

**Table 4:** Results of ICC within the group.

Sequence	Parameter	ICC (Con,1)	95% CI
SMS+SS-EPI (n = 48)	SNR	0.825	0.707 ~ 0.898
	CNR	0.810	0.685 ~ 0.889
	C	0.800	0.670 ~ 0.883
	ADC	0.875	0.788 ~ 0.928
RS-EPI (n = 52)	SNR	0.806	0.685 ~ 0.884
	CNR	0.773	0.635 ~ 0.863
	C	0.845	0.745 ~ 0.908
	ADC	0.858	0.766 ~ 0.916

Con represents consistency, and 1 represents a single measure.

**Table 5:** Comparison of objective evaluation indicators of image quality between two sets of DWI (mean ± SD).

Sequence	SMS+SS-EPI (n = 48)	RS-EPI (n = 52)	P value
SNR	57.65 ± 7.84	50.45 ± 6.56	<0.001
CNR	4.58 ± 0.75	4.16 ± 0.73	0.005
C	6.43 ± 1.06	6.32 ± 1.02	0.578
ADC	0.90 ± 0.23	0.87 ± 0.21	0.448

### 3.4 Diagnostic efficiency

**Figure 2:**
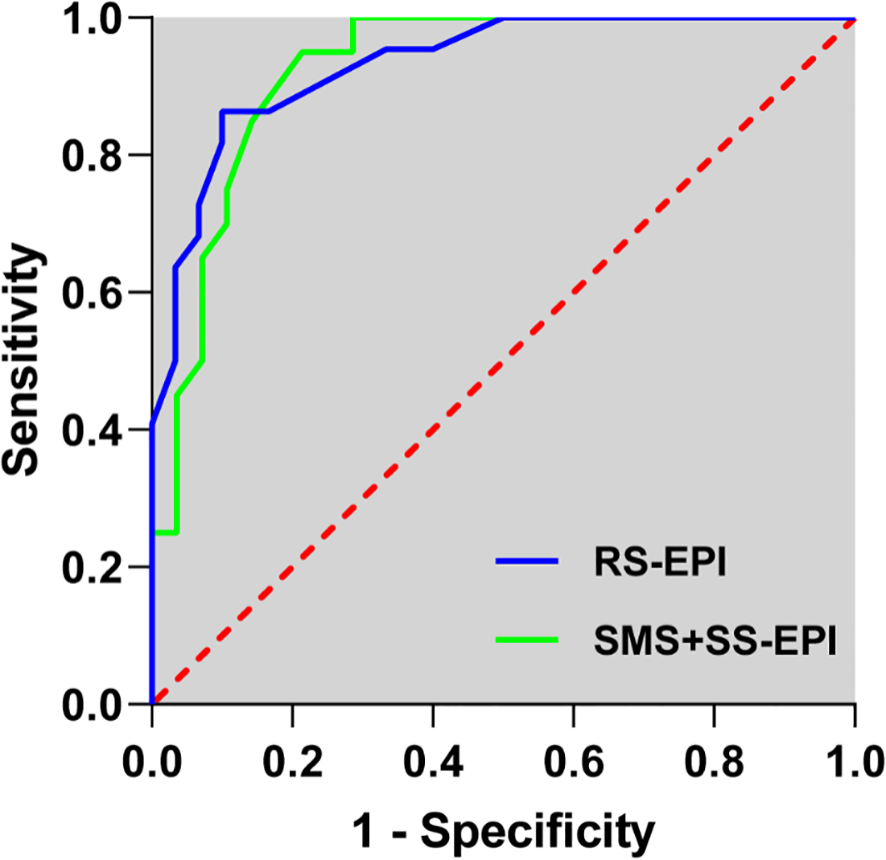
The receiver operating characteristic curve for the diagnosis of ADC values of prostate lesions in SMS+SS-EPI and RS-EPI sequence diffusion-weighted imaging.

Transperineal 12+X needle biopsy confirmed prostate cancer in 58 of 100 patients, including 28 in the SMS+SS-EPI group and 30 in the RS-EPI group. The AUC for diagnosing prostate lesions using ADC values in SMS+SS EPI sequence imaging was 0.925, with a sensitivity of 85.00% and specificity of 85.71%. The AUC for diagnosing prostate lesions using ADC values in RS-EPI imaging was 0.933, with a sensitivity of 86.36% and specificity of 83.33% (**Table 6**) (**Figure 2**). The AUC values of SMS+SS-EPI and RS-EPI were statistically equivalent (z=0.462, P = 0.644).

**Table 6:** Comparison of the effectiveness of ADC values in two sets of sequence imaging for diagnosing prostate cancer.

Classification	AUC (95% CI)	Sensitivity (%)	Specificity (%)	Youden index	P value
SMS+SS-EPI	0.925 (0.851-0.999)	85.00	85.71	0.707	<0.001
RS-EPI	0.933 (0.869-0.998)	86.36	83.33	0.697	<0.001

## 4 Discussion

This study represents the first systematic comparison of image quality and diagnostic performance between SMS+SS-EPI and RS-EPI in prostate DWI. Results indicate that SMS+SS-EPI offers significant advantages in scanning efficiency, while RS-EPI demonstrates superior subjective image quality assessment. Objective metric analysis revealed that SMS+SS-EPI demonstrated higher SNR and CNR, suggesting advantages in signal preservation and lesion contrast enhancement. No significant differences were observed between the two sequences in terms of ADC values, and their diagnostic performance for prostate cancer was statistically equivalent, indicating comparable clinical reliability in lesion characterization.

Prostate MRI routinely contains multipara metric sequences with a total duration of 30–40 min, which are poorly tolerated by patients and have frequent motion artifacts (27, 28). In this study, the DWI acquisition time of SMS+SS-EPI sequence was significantly shortened, which not only improves patient tolerance and reduces motion artifacts, but also enhances the examination throughput per unit of time, which is particularly suitable for the optimization of multi-parameter MRI examination process. In addition, rapid imaging sequences are more clinically useful for patients with pain or anxiety who cannot remain still for long periods of time (29, 30). The core challenge of the DWI-EPI sequence is magnetic susceptibility artifacts with T2 blurring. RS-EPI shortens the effective echo chain length by segmented sampling in the readout direction, significantly reducing phase accumulation error. It demonstrates superior geometric fidelity at the prostate-rectum and prostate-bladder air interfaces compared to SS-EPI (31, 32). Research findings indicate that RS-EPI significantly outperforms SMS+SS-EPI in anatomical distortion, image sharpness, and detail display scores (P<0.001). This suggests RS-EPI technology is particularly well-suited for anatomical regions like the prostate—which is close to the rectum and features numerous air-tissue interfaces—enabling superior preservation of anatomical integrity and lesion margin clarity. In scenarios prioritizing diagnostic accuracy, RS-EPI retains irreplaceable advantages. It is noteworthy that the overall image quality in the SMS+SS-EPI group still achieved a subjective average score of ≥3, indicating that the artifacts are acceptable and meet the minimum PI-RADS reading requirements. Although the subjective scoring favored RS-EPI, objective metrics revealed significantly higher SNR and CNR for SMS+SS-EPI. This may stem from SMS technology preserving signal intensity to some extent by simultaneously exciting multiple layers and utilizing coil sensitivity information for image reconstruction, unlike parallel imaging which suffers SNR degradation due to k-space undersampling (33, 34). The larger field of view and lower matrix employed by SMS+SS-EPI may also contribute to higher signal intensity to some extent, but at the cost of relatively reduced spatial resolution. This may explain its lower subjective ratings for detail display and sharpness. ADC values are important quantitative indicators for prostate cancer diagnosis (35, 36). The results of this study showed that there was no significant difference between the two sequences in terms of contrast and ADC values, indicating that SMS+SS-EPI has good reliability in retaining tissue diffusion information. Additionally, in the diagnosis of prostate cancer, ROC curve analysis based on ADC values for the two sequences showed AUC values of 0.925 and 0.933 for SMS+SS-EPI and RS-EPI, respectively. The diagnostic performance of the two sequences was statistically equivalent, with comparable sensitivities and specificities. This indicates that in lesion characterization, SMS+SS-EPI maintains its diagnostic value despite reduced image sharpness, further supporting the application potential of SMS technology in tumor DWI.

The findings of this study provide valuable guidance for clinical applications. The RS-EPI sequence offers advantages in subjective image quality perception, particularly in anatomical structure visualization and detail presentation. This is highly valuable in clinical scenarios requiring precise anatomical analysis. For instance, in prostate cancer staging, surgical planning, and radiotherapy target delineation, the RS-EPI sequence provides clearer anatomical information, thereby enhancing diagnostic and therapeutic accuracy. However, the SMS+SS-EPI sequence demonstrates significant advantages in lesion SNR and CNR, coupled with shorter scan times, making it more suitable for clinical scenarios requiring rapid prostate cancer detection. For instance, in emergency situations or examinations demanding high patient cooperation, the SMS+SS-EPI sequence can swiftly deliver diagnostic information, reducing patient wait times. Therefore, in practical applications, sequences should be selected flexibly based on clinical objectives.

### 4.1 Limitations

This study has several limitations. The relatively small sample size may affect the reliability and generalizability of the findings. As a retrospective study, selection bias and information bias may exist. Future studies should expand the sample size and strictly control study variables to enhance the accuracy and reliability of the results. Furthermore, this study only compared the image quality of SMS+SS-EPI and RS-EPI sequences in prostate cancer DWI. The combined application of other imaging sequences has not been thoroughly explored. Future research could investigate the combined use of multiple imaging sequences to improve the diagnostic accuracy of prostate cancer.

## 5 Conclusion

The SMS+SS-EPI and RS-EPI sequences each have distinct advantages and disadvantages in terms of image quality for prostate DWI. The RS-EPI sequence demonstrates superior subjective image quality, particularly in anatomical clarity and detail visualization. Conversely, the SMS+SS-EPI sequence offers advantages in lesion SNR and CNR, coupled with shorter scan times. Both sequences exhibit comparable performance in ADC value measurement and diagnostic efficacy, each possessing strong clinical utility. In clinical practice, imaging sequences should be selected judiciously based on specific diagnostic requirements and patient characteristics.

## Data Availability

The original contributions presented in the study are included in the article/supplementary material. Further inquiries can be directed to the corresponding author/s.
